# Comparative Study of Potassium Ion-Selective Electrodes with Solid Contact: Impact of Intermediate Layer Material on Temperature Resistance

**DOI:** 10.3390/molecules29235803

**Published:** 2024-12-09

**Authors:** Klaudia Morawska, Szymon Malinowski, Magdalena Wardak, Cecylia Wardak

**Affiliations:** 1 Department of Analytical Chemistry, Institute of Chemical Sciences, Faculty of Chemistry, Maria Curie-Sklodowska University, Maria Curie-Sklodowska Sq. 3, 20-031 Lublin, Poland; klaudia.morawska@mail.umcs.pl; 2 Faculty of Civil Engineering and Architecture, Lublin University of Technology, Nadbystrzycka Sq. 40, 20-618 Lublin, Poland; s.malinowski@pollub.pl; 3 Independent Public Health Care Center of the Ministry of Internal Affairs and Administration in Lublin, ul. Grenadierów 3, 20-331 Lublin, Poland; magdawardak2@gmail.com

**Keywords:** ion-selective electrode, temperature resistance, solid contact, conducting polymer, multi-walled carbon nanotubes, copper oxide nanoparticles, nanocomposite, perinone polymer

## Abstract

This paper presents a comparative study on the temperature resistance of solid-contact ion-selective electrodes, depending on the type of solid-contact material. Five types of potassium electrodes, with a valinomycin-based model membrane, were developed using different types of mediation layers, namely a conductive polymer (poly(3-octylthiophene-2,5-diyl) and a perinone polymer), multi-walled carbon nanotubes, copper(II) oxide nanoparticles, and a nanocomposite consisting of multi-walled carbon nanotubes and copper(II) oxide. We examined how the measurement temperature (10 °C, 23 °C, and 36 °C) affects the sensitivity, measurement range, detection limit, selectivity, as well as the stability and reversibility of the electrode potential. Electrodes modified with a nanocomposite (GCE/NC/ISM) and a perinone polymer (GCE/PPer/ISM) showed the best resistance to temperature changes. An almost Nernst response and a stable measurement range and the lowest detection limit values for each temperature were obtained for them. The introduction of mediation layers significantly improved the stability and potential reversibility of all the modified electrodes relative to the unmodified electrode (GCE/ISM). Still, it was the GCE/PPer/ISM and GCE/NC/ISM that stood out from the others, with stability of 0.11 and 0.12 µV/s for 10 °C, 0.05 and 0.08 µV/s for 23 °C, and 0.06 and 0.09 µV/s for 36 °C, respectively.

## 1. Introduction

**Figure 1:**
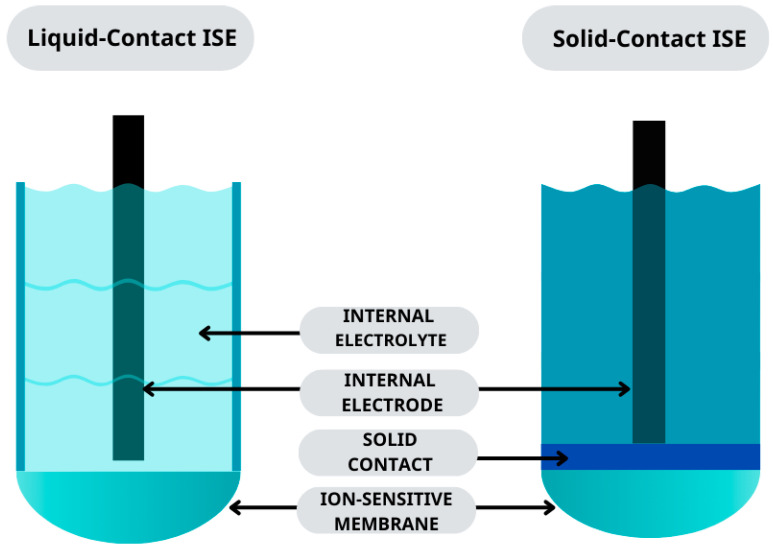
Design of liquid-contact ISE and solid-contact ISE.

For potentiometric measurements, mainly, two types of ion-selective electrodes (ISEs) are used, namely with liquid (LCISEs) or solid contact (SCISEs) (Figure 1). Increasingly, the first construction (with an internal electrolyte) is being replaced by electrodes with solid contact. The elimination of one element (the internal solution) revolutionized the world of electrochemistry back in the 1960s and 1970s [1].

Since then, many new possibilities have been created that were not possible with liquid-contact ISEs [2]. It became possible to miniaturize electrodes [3], so they could be created in ever newer and more convenient shapes (using various techniques, e.g., screen printing [4,5,6]), which enabled much easier manual operation (measurement in any position, no need to constantly refill the internal electrolyte) [7,8,9,10]. However, despite these conveniences, researchers struggled with the instability and poor reversibility of the potential of such electrodes, which was mainly due to the different conductivity at the membrane–internal electrode interface (the membrane has an ionic conductivity and the inner electrode, on the other hand, has an electron conductivity) [7,11]. An additional drawback is the possibility of the formation of an aqueous layer between these phases, which also contributes to generating greater potential drift over time [12]. The solution turned out to be the implementation of a layer with an ion-to-electron character (ion-to-electron conductivity), which is generally also selected for its hydrophobicity, stability, as well as its chemical inertness [13,14]. To date, the materials used to form the SC have included various conductive polymers [15,16,17,18], carbon nanomaterials [19,20,21,22], metal and metal oxide nanoparticles [23,24,25,26], ionic liquids [27,28,29,30], as well as composite materials [31,32,33,34,35]. All of these materials, due to their properties (through their use as a structural modifier, namely a solid contact), have made it possible to improve the performance of ISEs.

Nevertheless, it is not only the construction of the electrodes that affects their potential and the way they work. It is necessary to take into account several external factors which, to a greater or lesser extent, can interfere with the measurements and lead to the formation of various interferences and, consequently, to obtaining erroneous measurement results [36]. Among these, we must pay attention to the composition of the test solution (interferences due to the presence of ions or compounds other than the main ion), the choice of a suitable reference electrode, changes in pressure [37], and also changes in temperature [38,39]. This last parameter is closely related to the performance of ISEs because the temperature changes the electrode potential, according to the Nernst Equation (1):

(1) $$E=E^{0}+\frac{RT}{zF}lna$$

where: *E*—electrode potential; *E*^0^—standard potential; *R*—gas constant [8.314 J/(mol × K)]; *T*—temperature [K]; *z*—charge of the main ion to which the electrode is selective; *F*—Faraday’s constant [96,485 C/mol]; *a*—activity of the main ion [40,41]. The temperature change has the greatest effect on the sensitivity of the electrode (slope) [42,43], because the value increases or decreases proportionally to the increase or decrease in temperature, because:

(2) $$S=\frac{RT}{zF}$$

where: *S*—slope [mV/decade]. An increase in temperature increases the value of this coefficient and, thus, makes the electrode more sensitive to a change in the activity of the main ion (theoretically the value of the slope increases by about 2 mV/decade as a result of a temperature increase of 10 °C) [39,44,45]. Although it should be remembered that too high a temperature, or too rapid a change, can lead to measurement errors and also to the destruction of the electrode. Therefore, when developing a novel modification, the aim is to obtain an electrode with the most stable and reversible potential over time, regardless of the change in the measurement conditions, i.e., temperature, light, and environmental pH, etc.

In publications describing solid-contact ion-selective electrodes, the effects of factors such as the presence of gases [46], varying illumination [47], pH [48], and changes in redox potential [49], are often investigated. Typically, all measurements are carried out at room temperature [9,50,51] and there is very little information on the behavior of SCISEs under other temperature conditions [38,45]. Ion-selective electrodes are used in many areas (in situ environmental measurements, in vivo clinical measurements), where it is difficult and sometimes impossible to maintain room temperature during measurement. For this reason, in this paper, we focus on the influence of temperature, changes of which can have a significant impact on, among other things, the stability of the intermediate layer and the chemical stability of the membrane, which in turn will affect subsequent processes taking place around the membrane. High or extremely low temperatures can lead to the decomposition or deformation of the solid-contact material and some membrane components, or can contribute to changes in the membrane structure (in the worst case, to membrane destruction and separation from the substrate) [38] and changes in the equilibrium at the membrane–solution interface [52], which in turn can affect not only the sensitivity, but also the stability and reversibility of the electrode potential.

For this study, we selected materials that are widely known and have been successfully used as solid-contact materials, belonging to different material types, i.e., conductive polymers, carbon nanomaterials, metal oxide nanoparticles, and composite materials. However, electrodes based on these materials, although widely used and extensively studied, have not been tested for their temperature resistance. Therefore, we decided to analyze their applicability when subject to different temperature conditions.

In the current study, five different types of electrodes containing different solid-contact materials were developed: conductive polymers (poly(3-octylthiophene-2,5-diyl) (POT), a perinone polymer (PPer), carbon nanomaterials (multi-walled carbon nanotubes (MWCNTs)), nanoparticles (copper(II) oxide nanoparticles (CuONPs)), and a nanocomposite (MWCNTs and CuONPs nanocomposite). These electrodes were tested under different temperature conditions (10 °C, 23 °C, 36 °C) to investigate the effect of temperature on their basic analytical parameters: the limit of detection, measurement range, the slope of the characteristics, the stability and reversibility of the potential, and selectivity.

## 2. Results and Discussion

### 2.1. Potentiometric Response of SCISEs (Calibration Curves, etc.)

**Figure 2:**
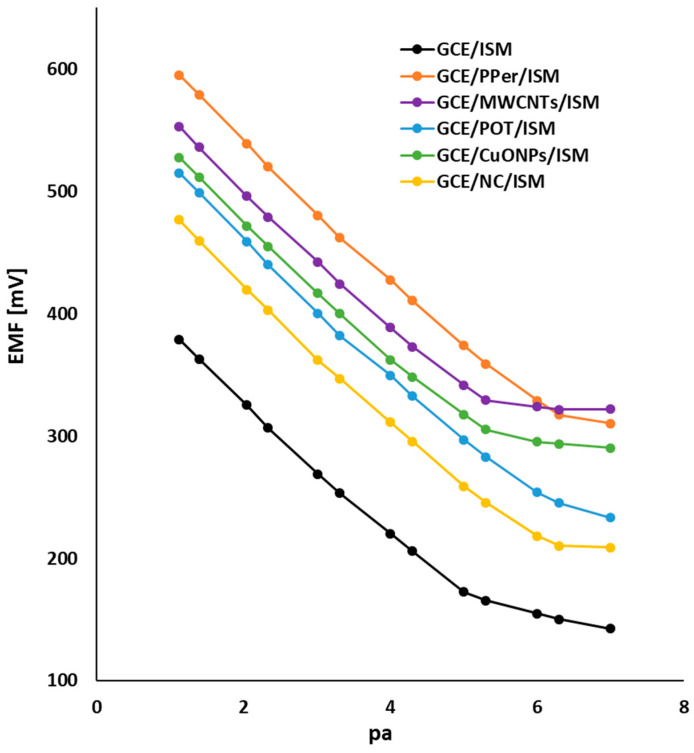
Characteristics of the electrodes determined at 23 °C.

**Figure 3:**
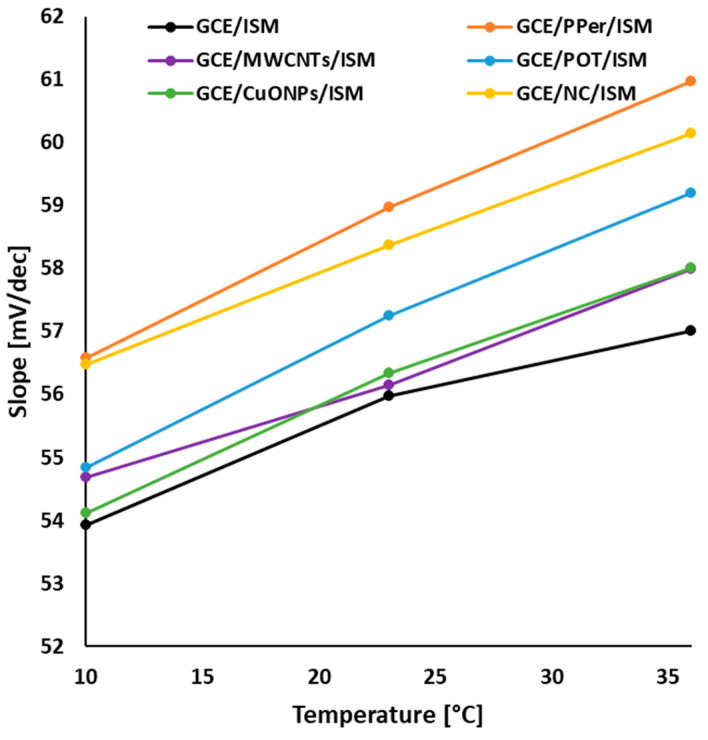
Slope of the characteristics determined for each electrode dependent on the temperature.

To determine the fundamental analytical parameters of the electrodes (which describe the quality of the individual ISEs), measurements were carried out to obtain the characteristics of the SCISEs at three different temperatures: 10 °C, 23 °C, and 36 °C. In regard to these measurements, not only the sensitivity, working range, and detection limits were assessed, but also how temperature affects a particular parameter and whether it has an unfavorable or even destructive effect on the electrodes’ lifespan. To create calibration curves of the electrodes, KNO_3_ solutions of 1 M, 1 × 10^−1^ M, 1 × 10^−2^ M, and 1 × 10^−3^ M were used (the measurement consisted of adding the appropriate amount of a specific concentration standard to obtain a curve in the concentration range 1 × 10^−1^–1 × 10^−7^ M). An exemplary set of calibration curves that was obtained at 23 °C, for each electrode, is shown in Figure 2. Based on the obtained results, the following parameters were determined, namely the slope of the characteristics (S) (Table 1), the detection limit (Table 2), and the linearity range (Table 3). Before analyzing the results, we focused on the estimation of the theoretical values of the slopes resulting from the Nernst Equation (1). Since our electrodes were tested in solutions containing a single ion, we can use the correlation described by Equation (2) to determine the theoretical slope values. Consequently, for temperatures of 10 °C, 23 °C, and 36 °C, the theoretical value of S is 56.18, 59.16, and 61.37 mV/decade, respectively. This means that as the temperature increases, the slope of the electrode will increase, which leads to greater sensitivity of the electrode to changes in the ion concentration (however, it should be noted that the temperature cannot be too high, which is dependent on the composition of the ion-sensitive membrane, the solid-contact material, and is dependent on the boiling, melting, or decomposition temperature of each component). The S values that were closest to the theoretical ones were obtained for the GCE/PPer/ISM and GCE/NC/ISM electrodes. The POT-modified electrode also performed very well in this regard. In the case of the other modifications using single solid-contact materials, i.e., MWCNTS and CuONPs, we obtained S values that gently deviated from those calculated using the Nernst equation, yet that increased in proportion with the temperature growth. Modifications of the electrodes with different solid-contact layers contributed to obtaining significantly better sensitivity at each temperature compared to the GCE/ISM (unmodified), for which the values were considerably lower than the theoretical value, and their changes with temperature deviated from the proportionality trend. Graphically, the dependence showing the effect of temperature on the slope is shown in Figure 3. As we can see, for the modified electrodes rectilinear sections of the curve occur, while for the GCE/ISM, we see a deviation from the rectilinear course of the curve. By analyzing the data in Table 2 and Table 3, it was found that temperature has little effect on the linearity and the detection limit of the electrodes. In the case of the first parameter (linearity range), for a temperature of 10 °C in regard to the GCE/MWCNTs/ISM, GCE/CuONPs/ISM, and GCE/ISM, we observe a narrowing of the working range of the electrodes (from 1 × 10^−5^–5 × 10^−2^ M to 1 × 10^−4^–1 × 10^−2^ M for the unmodified electrode and from 5 × 10^−6^–1 × 10^−1^ M to 1 × 10^−6^–1 × 10^−1^ M for the ISEs modified with CuONPs and MWCNTs), while for the other SCISEs, this parameter remains unchanged and equaled 5 × 10^−7^–1 × 10^−1^ M. The presence of a nanocomposite, a perinone polymer, and poly(3-octylthiophene-2,5-diyl), allowed us to obtain not only the widest working range, but also the lowest detection limits (below μM). Due to the fact that the temperature can change the membrane properties, such as the conductivity or chemical equilibrium at the membrane–solution interface, we can obtain shifted values in terms of the E^0^ parameter (which is why calibration is so important). The observation of this parameter is especially important for electrodes with polymeric membranes, which have a greater sensitivity to temperature than other membranes.

### 2.2. Stability and Reversibility of the Electrode Potential

**Figure 4:**
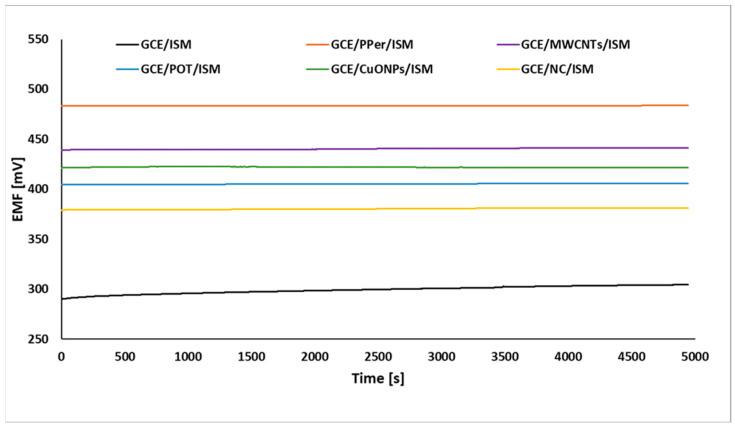
Short-term stability of all the electrodes determined at 23 °C.

**Figure 5:**
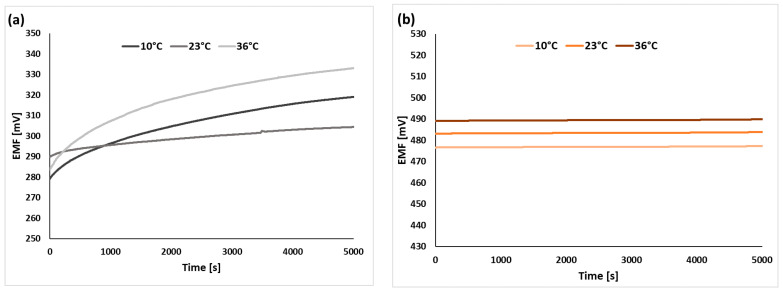

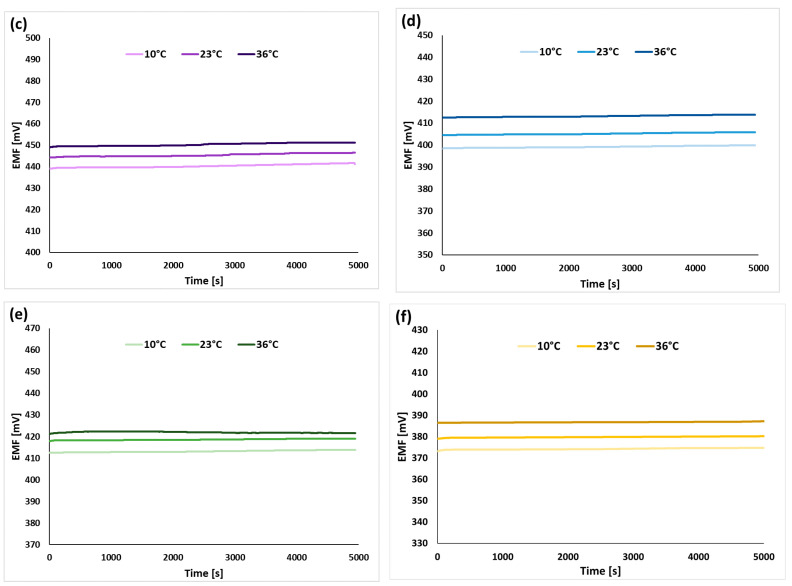
The comparison of the potential stability of GCE/ISM (**a**), GCE/PPer/ISM (**b**), GCE/MWCNTs/ISM (**c**), GCE/POT/ISM (**d**), GCE/CuONPs/ISM (**e**), GCE/NC/ISM (**f**), for each temperature.

The stability and reversibility of the electrode potential are among the most important parameters of ion-selective electrodes, affecting the accuracy of the analysis results. When electrodes are used at different temperatures, these parameters are significantly influenced not only by the chemical stability of the membrane (the possible decomposition of some components), but also by the selection of a suitable modifying component that will exhibit thermal resistance over a wide temperature range. When we say modifying component, we mean a solid-contact material that mainly provides ion-to-electron conductivity between the membrane (in this case, potassium) and the inner electrode (glassy carbon), thus improving the stability and reversibility of the ISE potential. To determine the stability of the tested electrodes (modified and conventional GCE covered only with a membrane), the potential was measured continuously for 1.5 h in a 1 × 10^−3^ M KNO_3_ solution at three different temperatures (10 °C, 23 °C, and 36 °C) (the short-term stability of the tested electrodes, determined at 23 °C, is presented in Figure 4). In addition, a combination of the stability for each temperature was developed for the unmodified electrode (Figure 5a) and those modified with a perinone polymer (Figure 5b) (this electrode shows the best stability of all those presented), MWCNTs (Figure 5c), POT (Figure 5d), CuONPs (Figure 5e), and the nanocomposite (Figure 5f). From the graphs presented, we can already see, with the naked eye, a huge difference in the stability aspect of these electrodes. The unmodified electrode was characterized by high instability, which significantly deteriorated both at temperatures below room temperature (10 °C) and those higher (36 °C) than room temperature, while the GCE/PPer/ISM and GCE/NC/ISM electrode, regardless of the temperature, showed excellent stability (the other electrodes also had satisfactory stability, but was weaker than that for both the SCISEs). The stability shown in the graphs was also described by calculating the potential drift over time (the difference between the initial and final potential over time). The values of this parameter, expressed in μV/s, determined for each electrode (at each temperature), are shown in Table 4. Based on the results achieved, it was concluded that the applied mediation layer materials showed very satisfactory temperature stability. The lowest values of the potential drift were obtained for the electrode modified with a perinone polymer and a nanocomposite of copper(II) oxide and multi-walled carbon nanotubes. Compared to the unmodified electrode, for the GCE/PPer/ISM electrode, 66, 73, and 158 times better stability was obtained, respectively, for the temperatures 10 °C, 23 °C, and 36 °C. For the GCE/NC/ISM electrode, the drift values were lower than for the GCE/ISM at 10 °C, 23 °C, and 36 °C, by 60, 45, and 105 times, respectively. For the other solid-contact materials, equally satisfactory values in terms of the potential drift over time were obtained, but deviated from those for the best-performing electrodes.

**Figure 6:**
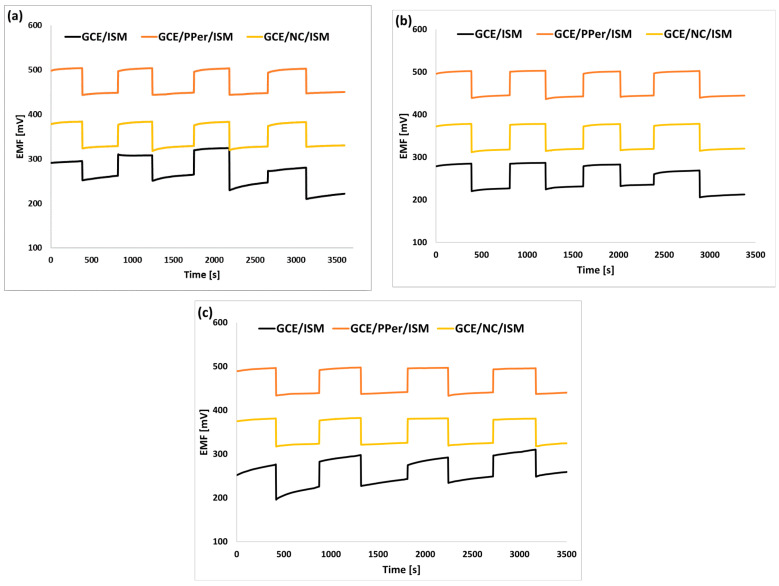
Reversibility of the electrode potential for the GCE/ISM, GCE/PPer/ISM, and GCE/NC/ISM electrodes determined at 10 °C (**a**), 23 °C (**b**), and 36 °C (**c**).

As mentioned above, electrode potential reversibility is as important as its stability. To check whether the electrodes we developed showed good potential reversibility (regardless of temperature), a series of tests were carried out. These tests involved measuring the electrode potential using alternate potassium nitrate(V) solutions of 1 × 10^−3^ M and 1 × 10^−4^ M (four times) at three different temperatures (10 °C, 23 °C, and 36 °C). The results of the measurements for the unmodified electrode and the GCE/PPer/ISM and GCE/NC/ISM electrodes are shown in Figure 6a–c (for temperatures of 10 °C, 23 °C, and 36 °C). Then, based on the acquired results, the average values of the electrode potential with standard deviations for a particular concentration were calculated, and the results are detailed in Table 5. It is concluded from the analysis of the results that, also in this case, the use of the nanocomposite and the perinone polymer turned out to be the best solution, which allowed us to attain a reversible potential regardless of the measurement conditions (the lowest values of the standard deviation (SD) from the average value of the potential for both concentrations were obtained for these electrodes, the SD values for the 1 × 10^−3^ M and 1 × 10^−4^ M KNO_3_ solutions, for these electrodes, were almost 20 times better than for the unmodified electrode). The electrodes modified with MWCNTs, CuONPs, or POTs performed not much worse in regard to this aspect. All the modified electrodes showed reversible electrode potential for all the measurement conditions, which was not the case for the GCE/ISM electrode.

### 2.3. Selectivity

To determine the potentiometric selectivity coefficient, measurements were carried out in a solution of the main ion (KNO_3_) and a solution of interfering ions. The measurements for the main ion were carried out for concentrations from 1 × 10^−6^ to 1 × 10^−1^ M (in the same way as the calibration curves described in the subsection on the potentiometric response of the SCISEs). After determining the calibration curve for main ion (K^+^) with the addition of the standards, solutions of interfering ions were prepared at concentrations of 1 × 10^−1^ M and 1 M. The interfering ions were sodium, magnesium, and calcium ions. The calibration curves for the interferents were plotted in the same way as for the main ion in the concentration range of 1 × 10^−4^–1 × 10^−1^ M. Based on the obtained results, the values of the logarithmic values of selectivity coefficient were calculated (Table 6). The modified electrodes showed a slightly better selectivity relative compared to the unmodified electrode. In most cases, better selectivity was obtained at extreme temperatures than at room temperature.

## 3. Materials and Methods

### 3.1. Materials

Membrane components: valinomycin—potassium ionophore (Fluka, Pułtusk, Poland); potassium tetrakis-parachlorophenyl borate (KTpClB) (Sigma-Aldrich, Pułtusk, Poland); poly(vinyl chloride) (PVC) (Sigma-Aldrich); di-2-ethylhexyl sebacate (DOS) (Fluka). The materials used to apply the perinone polymer layer: perinone monomers (Per) (as a mixture of anti-isomer benzo[lmn]diperimidino[2,1-b:2′,1′-i][3,8]phenanthroline-7,18-dione and syn-isomer benzo[lmn]diperimidino [2,1-b:1′,2′-j][3,8]phenanthroline-5,8-dione) (the synthesis process is described in paper [15]), dichloromethane (POCH); tetrabutylammonium hexafluorophosphate (Sigma-Aldrich). The materials used to obtain the intermediate layers: MWCNTs (length 3–6 μm, outer diameter 10 nm ± 1 nm, inner diameter 4.5 nm ± 0.5 nm), CuONPs (particles size < 50 nm) (Sigma-Aldrich), POT (Aldrich, Pułtusk, Poland). All the salts used for the potentiometric measurements are high-purity salts for analysis (Fluka).

### 3.2. Measurements

The potentiometric measurements were carried out in a system consisting of a working electrode (potassium ion-selective electrodes with different types of solid contact) and a reference electrode (silver/silver chloride electrode with a double junction (6.0750.100, Metrohm, Herisau, Switzerland)). All the measurements were carried out using a thermostat (LAUDA, Ecoline Staredition RE104, Lauda-Königshofen, Germany) at temperatures of 10 °C, 23 °C, and 36 °C, in solutions mixed using a magnetic stirrer.

### 3.3. Preparation of Ion-Sensitive Membranes and Ion-Selective Electrodes

For the preparation of the potassium ion-selective electrodes, ISEs with a glassy carbon inner electrode, with a diameter of 0.3 cm, were used (three copies were prepared for each electrode type). All the electrodes used in the study were pre-treated before being modified. The pre-treatment was conducted in several stages and presented as follows: (1) GCE polishing using wetted Al_2_O_3_ on felt, (2) rinsing the electrodes with distilled water and then placing them in an ultrasonic bath to get rid of any impurities (mainly Al_2_O_3_), and (3) rinsing the electrodes again with distilled water and degreasing their surfaces by immersing them in a THF solution. After pre-treatment, each group of three electrodes (except for the electrodes acting as control electrodes, according to which an ion-sensitive membrane was applied during the last step) was modified by applying a mediation layer to the surface of the cleaned GCE. In most cases, solutions containing the appropriate concentration of transducer media were prepared to apply the solid-contact layer (the exception here is the perinone polymer, which was applied by cyclic voltammetry from a saturated electrolyte solution using potentiometric polymerization). The solutions containing the modifier materials were then homogenized in an ultrasonic bath for one hour. In this way, five different types of potassium ion-selective electrodes were developed: (1) an electrode with solid contact in the form of a perinone polymer (GCE/PPer/ISM), the intermediate layer was created via potentiodynamic polymerization of the PPer precursors from an electrolyte solution (the process of PPer polymer application is described in detail in paper [15]). (2) An electrode with an intermediate layer constituted by a POT (GCE/POT/ISM). The POT was placed by spotting 10 μL of a 25 mM polymer solution in chloroform onto the electrode surface. Then, the solvent was left to evaporate for 24 h. (3) An electrode with a mediation layer constituted by multi-walled carbon nanotubes (GCE/MWCNTs/ISM). To deposit the multi-walled carbon nanotubes, a suspension of MWCNTs, at a concentration of 5 mg/mL, was prepared. For this purpose, 15 mg of MWCNTs was dispersed in 3 mL of THF. Then, 6 μL of this suspension was applied to the electrode substrate and the solvent was left to evaporate. (4) Electrodes with solid contact in the form of copper(II) oxide nanoparticles (GCE/CuONPs/ISM). Initially, a suspension of CuONPs in THF was prepared (3 mg of nanomaterial per 1 mL of solvent). The suspension prepared in this way was spotted, with a volume of 30 μL, on the electrode contact and then the THF was left to evaporate. (5) Electrodes modified with an intermediate layer made of a nanocomposite of multi-walled carbon nanotubes and copper(II) oxide (GCE/NC/ISM). Moreover, 15 μL of nanocomposite suspension (3 mg of NC per 1 mL of THF) was drop-casted onto the electrode substrate and the solvent was left to evaporate. The preparation and application of the composite suspension are described in detail in paper [49].

**Figure 7:**
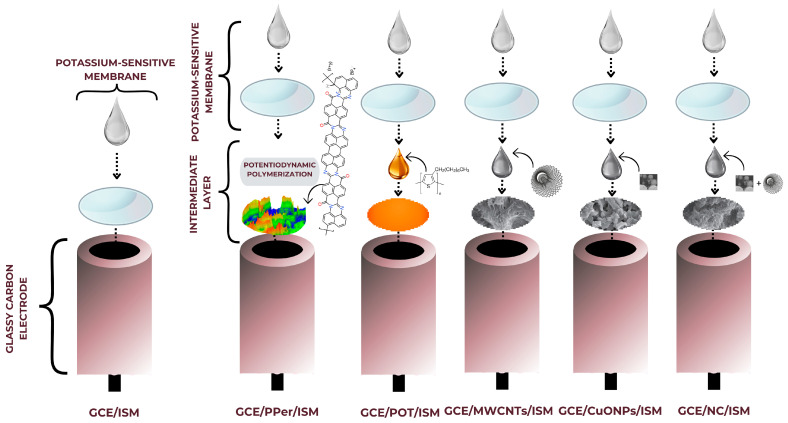
Schematic diagram on the preparation of the electrodes.

In order to provide each of the prepared electrodes with selectivity towards potassium ions, a membrane mixture consisting of DOS (64%), PVC (32%), valinomycin (3%), and KTpClB (1%) was prepared. The composition as prepared (0.3 g) was dissolved in tetrahydrofuran (3 mL). On each of the appropriately prepared electrodes (as described above), 50 μL of the mixture was spotted in a series of three spots, every 30 min. The electrodes were vertically positioned to let the solvent evaporate from the membrane and then they were placed in a conditioning solution (1 × 10^−3^ M KNO_3_) for two days. A diagram of the electrode preparation is shown in Figure 7.

## 4. Conclusions

This paper presents the effect of temperature on the analytical parameters of ion-selective electrodes modified with various solid-contact materials, from different material classes. Since all the electrodes tested had the same valinomycin-based ion-sensitive membrane, the differences observed are due to differences in the properties of the solid-contact materials used. The mediation layers were made using a perinone polymer, poly(3-octylthiophene-2,5-diyl), multi-walled carbon nanotubes, copper oxide nanoparticles, and a nanocomposite consisting of MWCNTs and CuONPs. The study of such relationships is of significant important due to the possibility of using electrodes in environmental studies, where conditions are often far from those found in a laboratory, which is why it is necessary to study analytical devices in terms of their resistance to different temperatures, for example. The performance of electrodes is closely related to the stability and reversibility of their potential. A more robust and stable electrode response, characterized by a high slope (sensitivity), a low detection limit, a broad linear range, and a stable E^0^ parameter, leads to results that are highly reproducible and consistent, demonstrating improved stability and the reversibility of the electrode potential. The presence of an intermediate layer favored the analytical performance of SCISEs and helped stabilize their readings at different temperatures (10 °C, 23 °C, and 36 °C). The unmodified electrode showed high instability and a rather poor response (sub-Nernst). Among the presented electrodes, the GCE/PPer/ISM and GCE/NC/ISM electrodes showed the greatest resistance to temperature changes. For these electrodes, the best sensitivity (closest to the theoretical values), the widest, yet stable, linearity range at any temperature, and the lowest detection limit values, were obtained. The electrodes modified with the perinone polymer and the nanocomposite also showed extremely good stability and potential reversibility (this is clear evidence that the solid-contact materials used, as well as the components of the ion-sensitive membranes, are not distorted/damaged by their exposure to extreme temperatures, which is an extremely significant advantage).

The comparative results presented here provide new information on the temperature-dependent properties of ion-selective electrodes, based on different solid-contact materials. This information will enable a more appropriate choice of electrode for specific analytical tasks.

## Figures and Tables

**Table 1:** Slope of the characteristics (% value of the theoretical value) determined at 10 °C, 23 °C, and 36 °C [mV/decade].

Temperature	GCE/ISM	GCE/PPer/ISM	GCE/MWCNTs/ISM	GCE/POT/ISM	GCE/CuONPs/ISM	GCE/NC/ISM
10 °C	53.93 (95.99%)	56.57 (100.69%)	54.68 (97.33%)	54.84 (97.61%)	54.12 (96.33%)	56.48 (100.53%)
23 °C	55.27 (93.42%)	58.97 (99.68%)	56.14 (94.90%)	57.45 (97.11%)	56.44 (95.40%)	58.37 (98.66%)
36 °C	57.12 (93.07%)	60.97 (99.34%)	57.98 (94.47%)	59.19 (96.45%)	58.01 (94.53%)	60.14 (98.00%)

**Table 2:** Limit of detection calculated for 10 °C, 23 °C, and 36 °C [M].

Temperature	GCE/ISM	GCE/PPer/ISM	GCE/MWCNTs/ISM	GCE/POT/ISM	GCE/CuONPs/ISM	GCE/NC/ISM
10 °C	1.75 × 10^−5^	4.89 × 10^−7^	0.51 × 10^−6^	9.21 × 10^−7^	0.97 × 10^−6^	4.99 × 10^−7^
23 °C	8.94 × 10^−6^	4.77 × 10^−7^	4.21 × 10^−6^	7.87 × 10^−7^	2.54 × 10^−6^	4.41 × 10^−7^
36 °C	9.85 × 10^−6^	4.65 × 10^−7^	4.01 × 10^−6^	7.72 × 10^−7^	4.98 × 10^−6^	4.29 × 10^−7^

**Table 3:** Linearity range of the electrodes determined at 10 °C, 23 °C, and 36 °C [M].

Temperature	GCE/ISM	GCE/PPer/ISM	GCE/MWCNTs/ISM	GCE/POT/ISM	GCE/CuONPs/ISM	GCE/NC/ISM
10 °C	1 × 10^−4^–1 × 10^−2^	5 × 10^−7^–1 × 10^−1^	1 × 10^−6^–1 × 10^−1^	1 × 10^−6^–1 × 10^−1^	1 × 10^−6^–1 × 10^−1^	5 × 10^−7^–1 × 10^−1^
23 °C	1 × 10^−5^–5 × 10^−2^	5 × 10^−7^–1 × 10^−1^	5 × 10^−6^–1 × 10^−1^	1 × 10^−6^–1 × 10^−1^	5 × 10^−6^–1 × 10^−1^	5 × 10^−7^–1 × 10^−1^
36 °C	1 × 10^−5^–5 × 10^−2^	5 × 10^−7^–1 × 10^−1^	5 × 10^−6^–1 × 10^−1^	1 × 10^−6^–1 × 10^−1^	5 × 10^−6^–1 × 10^−1^	5 × 10^−7^–1 × 10^−1^

**Table 4:** Drift of the potential over time, calculated for each temperature [µV/s].

Temperature	GCE/ISM	GCE/PPer/ISM	GCE/MWCNTs/ISM	GCE/POT/ISM	GCE/CuONPs/ISM	GCE/NC/ISM
10 °C	7.28	0.11	0.95	0.38	0.78	0.12
23 °C	3.65	0.05	0.70	0.56	0.82	0.08
36 °C	9.51	0.06	0.68	0.44	0.91	0.09

**Table 5:** Mean potential values together with the standard deviation (n = 4) for each electrode measured at three different temperatures [mV].

Temperature	Solution [M]	GCE/ISM	GCE/PPer/ISM	GCE/MWCNTs/ISM	GCE/POT/ISM	GCE/CuONPs/ISM	GCE/NC/ISM
10 °C	1 × 10^−3^	300.11 ± 19.75	501.22 ±1.01	470.55 ± 1.89	413.08 ± 1.92	425.57 ± 2.01	381.22 ± 1.14
	1 × 10^−4^	243.63 ± 19.94	445.09 ± 1.27	415.59 ± 2.26	358.41 ± 2.41	370.24 ± 2.22	326.76 ± 1.57
23 °C	1 × 10^−3^	279.11 ± 8.65	500.61 ± 0.96	467.49 ± 1.26	418.42 ± 1.17	427.21 ± 1.54	376.36 ± 1.11
	1 × 10^−4^	224.59 ± 10.54	441.79 ± 1.13	413.33 ± 1.49	366.06 ± 1.39	373.58 ± 1.77	317.82 ± 1.24
36 °C	1 × 10^−3^	286.53 ± 15.75	495.38 ± 0.88	471.25 ± 2.14	417.68 ± 1.30	426.58 ± 1.98	380.19 ± 1.12
	1 × 10^−4^	237.36 ± 17.93	436.74 ± 0.93	418.21 ± 2.25	362.33 ± 1.31	372.49 ± 2.41	322.12 ± 0.99

**Table 6:** Logarithmic values of the selectivity coefficient of all electrodes at 10 °C, 23 °C, and 36 °C.

Interfering Ion	Temperature	GCE/ISM	GCE/PPer/ISM	GCE/MWCNTs/ISM	GCE/POT/ISM	GCE/CuONPs/ISM	GCE/NC/ISM
Na^+^	10 °C	−6.08	−6.56	−6.17	−6.49	−6.14	−6.58
	23 °C	−4.17	−4.57	−4.35	−4.55	−4.32	−4.55
	36 °C	−4.05	−4.85	−4.13	−5.30	−4.08	−5.01
Ca^2+^	10 °C	−5.28	−5.91	−5.39	−5.90	−5.74	−5.97
	23 °C	−3.83	−4.27	−3.98	−4.20	−4.01	−4.22
	36 °C	−6.08	−6.56	−6.17	−6.49	−6.14	−6.58
Mg^2+^	10 °C	−4.17	−4.57	−4.35	−4.55	−4.32	−4.55
	23 °C	−4.05	−4.85	−4.13	−5.30	−4.08	−5.01
	36 °C	−5.28	−5.91	−5.39	−5.90	−5.74	−5.97

## Data Availability

Data are available on request from the author.
